# Supplementary material for: Mortality in Pedigrees with Acute Intermittent Porphyria
Source: Life (Basel). 2022 Dec 8;12(12):2059. doi: 10.3390/life12122059 (PMC9785653; doi:10.3390/life12122059)
Supplement: Supplementary file 1 [file life-12-02059-s001.zip › life-1996072-supplementary.pdf]

**Appendix A: Supplementary figures S1-S3. Explanation of pedigree data collection procedure using an example pedigree.**

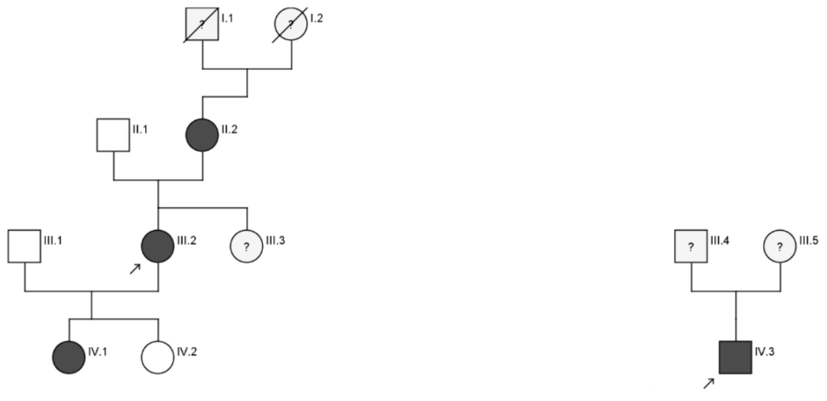


**Supplementary Figure S1. Step 1: Example pedigrees based on medical records only.**

---

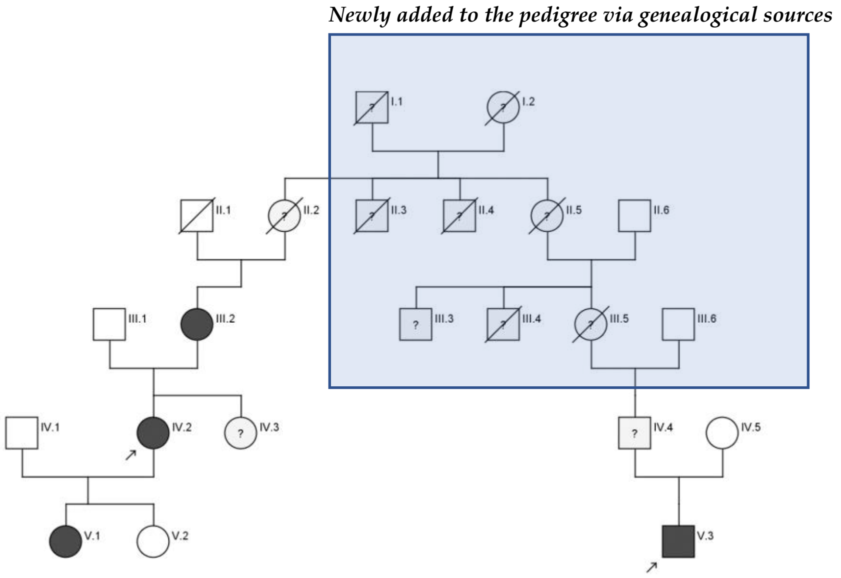


**Supplementary Figure S2. Step 2: Progression of the example pedigree after using publicly available genealogical sources.**

---

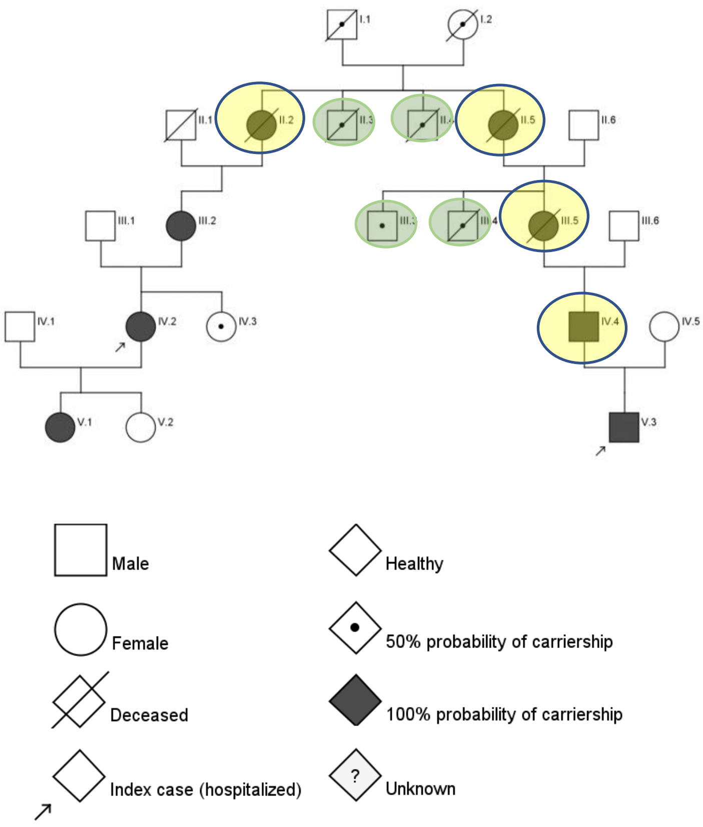


**Supplementary Figure S3. Step 3: Final example pedigree following confirmation with Dutch governmental records.**

----------------------------------------------------------------------------------------------------------------------------

**Legend Figures S1-S3**

Abbreviations. HMBS, hydroxymethylbilane-synthase. Figure S3 shows two (fictional) pedigrees which share the identical pathogenic *HMBS* variant; both families include hospitalised cases for acute porphyric attacks but are seemingly unrelated. Through genealogical research it was demonstrated that the pedigrees share a common ancestor (figure S2). After confirming dates of birth and death with Dutch governmental records we can now determine the probability that an individual is heterozygous in the final pedigree (figure S3). Parents, grandparents, great-grandparents and other family members who directly link both pedigrees together could be inferred to have passed on the variant to the next generation and were given a 100% probability to be heterozygous, even though testing is impossible, these are the individuals with the larger yellow circles, in this example 4 individuals with 100% probability . All other siblings and children connected to these newly identified heterozygotes were then also included with a 50% probability of heterozygosity, these are the individuals with the smaller light green circles, in this example 4 individuals with 50% probability.

Pedigrees graphs were made using CeGat Pedigree Chart Designer (© CeGaT GmbH, 2012-2020).
